# Impact of peri-urban pig farms on mosquito community structure in Yogyakarta, Indonesia

**DOI:** 10.1016/j.crpvbd.2025.100310

**Published:** 2025-08-20

**Authors:** Atikah Fitria Muharromah, Raden Roro Upiek Ngesti Wibawaning Astuti, Kozo Watanabe

**Affiliations:** a Center for Marine Environmental Studies (CMES), Ehime University, Bunkyo-cho 3, Matsuyama, Ehime, 790-8577, Japan; b Graduate School of Science and Engineering, Ehime University, Bunkyo-cho 3, Matsuyama, Ehime, 790-8577, Japan; c Department of Tropical Biology, Faculty of Biology, Universitas Gadjah Mada, Yogyakarta, 55281, Indonesia

**Keywords:** Mosquito vectors, Japanese encephalitis, Livestock, Yogyakarta

## Abstract

Livestock-keeping in peri-urban areas might influence mosquito-borne diseases by attracting more mosquitoes or by diverting mosquitoes from feeding on humans. In this study, we compared the mosquito community structure across pig farms, human settlements around pig farms, and human settlements without pig farms in peri-urban areas of Yogyakarta. We collected mosquitoes using ultraviolet light traps from three large pig farms (10 traps), 120 human settlements near pig farms (20 traps), and 120 human settlements without pig farms (20 traps) in Yogyakarta. The adult mosquitoes were morphologically identified using taxonomic keys. Polymerase chain reaction was used only to identify damaged and unidentified mosquito specimens using the cytochrome *c* oxidase subunit 1 gene marker. A total of 2253 adult mosquitoes (1663 females and 590 males) belonging to 26 species were collected from large pig farms, human settlements near pig farms, and human settlements without pig farms. Permutational multivariate analysis of variance revealed significant differences in mosquito community structure between the three areas (*R*^2^ = 0.66, *P* = 0.001). Large pig farms had higher mosquito diversity (26 species) than human settlements near pig farms (17 species) and human settlements without pig farms (10 species), with the highest number of Japanese encephalitis vector, *Culex tritaeniorhynchus* (381 individuals) collected in large pig farms and some zoophilic mosquitoes (e.g. *Anopheles vagus*, *An. aconitus*, *An. barbirostris*, *Mansonia uniformis*, *Mn. annulata*, *Cx. tritaeniorhynchus*, *Cx. hutchinsoni*, and *Mimomyia luzonensis*) found in human settlements near pig farms. Artificial containers for animal drinking were found to be breeding sites for *Aedes aegypti*, *Ae. albopictus*, and *Culex quinquefasciatus* in large pig farms. Raising pigs near human settlements increases the diversity and density of mosquito species, as demonstrated in our study, which also identifies pig farms as potential breeding sites for mosquitoes and highlights the higher risk of emergence of mosquito-borne diseases. Our data highlight the increased risk of Japanese encephalitis (JE) transmission in areas near pig farms and suggest that livestock relocation from human settlements and management practices to control mosquito breeding sites in pig farms in peri-urban areas could reduce the risk of disease outbreaks.

## Highlights

• Mosquitoes belonging to 26 species were collected from large pig farms and human settlements close to or without pig farms.
• Pig farms located near human settlements attracted a broader range of species, thus altering mosquito community structure.
• 22 mosquito species had higher CPUE values in large pig farms than in human settlements.
• A high density of the Japanese encephalitis vector, *Culex tritaeniorhynchus*, was recorded in large pig farms.
• The study highlights the increased risk of mosquito-borne diseases in peri-urban areas adjacent to pig farms.

## Graphical abstract

**Figure:**
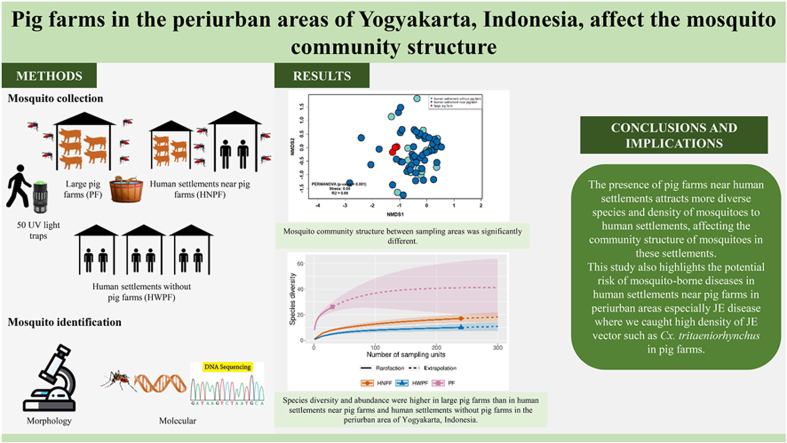

## 1 Introduction

The close proximity of livestock and human settlements may increase the risk of mosquito-borne infections. Communities living in peri-urban areas in developing Asian countries tend to raise livestock to supplement their income and diet (Ushimaru et al., 2024). Livestock are kept close to homes due to scarcity of land in peri-urban areas, which commonly consists of mosquitoes with moderately high species diversity (Johnson et al., 2020) that may be influenced by the presence of urban- and rural-related mosquitoes, such as *Aedes* spp., *Anopheles* spp., and *Culex* spp. (Johnson et al., 2020; Ombugadu et al., 2022). Therefore, the presence of animals in the peri-urban area increases interactions among animals, mosquitoes, and humans, facilitating the spread of urban- (e.g. dengue and chikungunya) and rural-related (e.g. malaria and filariasis) mosquito-borne diseases, including zoonotic diseases (e.g. Japanese encephalitis (JE) and zoonotic malaria), in peri-urban areas (Waage et al., 2022).

The epidemiological significance of livestock proximity to human settlements lies in how these animals influence mosquito abundance, diversity, and transmission dynamics. The presence of livestock can alter mosquito community composition and abundance due to species-specific feeding preferences. Livestock can increase mosquito abundance by providing more plentiful or easily accessible blood sources (e.g. *Psorophora columbiae*) (Kuntz et al., 1982; Service, 1991) and by creating more mosquito breeding sites by providing the feces-polluted standing water (e.g. *Culex quinquefasciatus*) (Rajagopalan et al., 1990; Service, 1991). In Tanzania, the presence of livestock has been shown to change the species composition of *Anopheles* spp., which can impact malaria transmission dynamics (Mayagaya et al., 2015). In Vietnam, livestock farming has been linked to an increase in the number of vectors for JE virus, specifically *Culex tritaeniorhynchus* (Lindahl et al., 2012). In Ethiopia, the presence of livestock attracted more zoophilic mosquitoes, which in turn led to increased human-bite rates and potential disease transmission risks (Seyoum et al., 2002). Moreover, studies in Vietnam and China have found that *Culex* spp. and *Anopheles* spp. mosquitoes were more abundant in human settlements with pig farms, suggesting that livestock presence is associated with higher mosquito populations in these regions (Ren et al., 2017; Nguyen-Tien et al., 2021a, 2021b; Pham-Thanh et al., 2022). These studies underscore the regional differences in how livestock can influence mosquito populations and disease transmission, which is crucial for understanding the local risks of vector-borne diseases. The majority of research on how pig farms affect mosquito communities has concentrated on certain mosquito species that are likely to be impacted, with little attention paid to the larger mosquito community. Notably, studies examining the effects of livestock farming on mosquito community structure at the species level are scarce.

Based on feeding behavior, mosquitoes can be categorized into those that prey primarily on animal hosts (zoophilic), human hosts (anthropophilic), or both (opportunistic) (Takken and Verhulst, 2013). Because opportunistic mosquitoes have a wider range of hosts (Busula et al., 2015), they play an important role in transmitting diseases between hosts, for example, from animal to human, human to animal, and animal to other animals. Mixed livestock and human settlements may provide mosquitoes with opportunities to feed on blood from animals and humans. This may facilitate the presence of opportunistic mosquitoes, the co-occurrence of zoophilic and anthropophilic species, and greater species diversity of the mosquito community than that in human settlements without livestock.

The objective of this study was to compare the mosquito community structure among pig farms, human settlements around pig farms, and human settlements without pig farms in peri-urban areas of Yogyakarta, Indonesia. Although Yogyakarta is one of urbanized cities in Indonesia, many residents on its outskirts continue to raise their livestock using traditional ways, often placing their animals close to their homes. We hypothesized that the presence of pig farms within human settlements influences mosquito community composition, driven by species-specific feeding preferences. We predicted that pigs kept near humans would attract a mix of zoophilic and anthropophilic mosquitoes due to the simultaneous availability of animal and human hosts in close proximity.

## 2 Materials and methods

### 2.1 Study site and mosquito collection

**Fig. 1:**
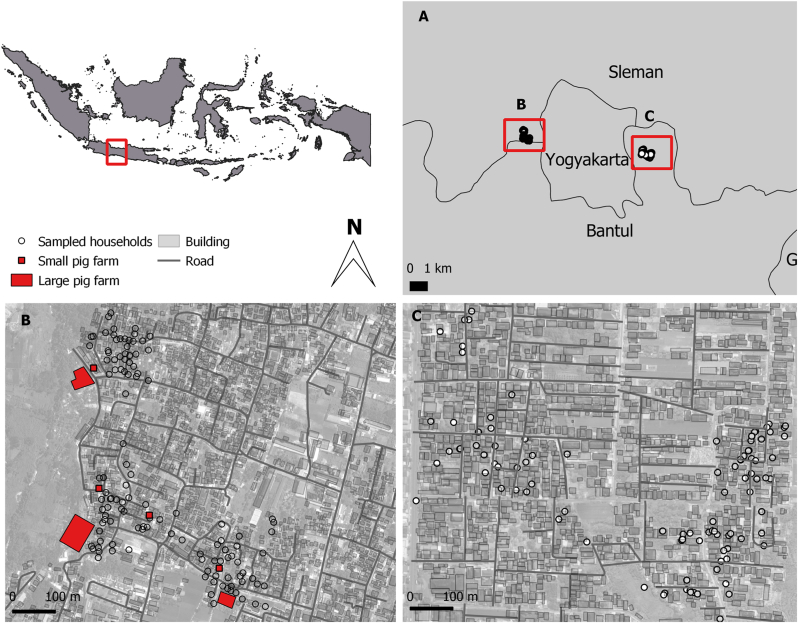
Sampling sites at the peri-urban areas of Special Region of Yogyakarta Province, Indonesia (**A**). The sampling sites consist of human settlements near pig farms in Ngestiharjo village (**B**) and human settlements without pig farms in Banguntapan village (**C**).

Mosquitoes were collected during the rainy season in two villages, Ngestiharjo and Banguntapan, Bantul District, Special Region of Yogyakarta Province, Indonesia (Fig. 1), using mosquito ultraviolet (UV) light traps (Krisbow, Indonesia). Human settlements in Ngestiharjo village are situated near large pig farms (less than 1 km away). Several households in Ngestiharjo village kept their own livestock, housing up to 22 pigs either behind or in front of their homes. Farmers tended to raise pigs collectively in certain areas within the village. We defined the collective pig farms as large pig farms and individual household pig farms as small pig farms. The distance between the large farms is about 500–700 m. By contrast, human settlements in Banguntapan village had no pig farms, making this village a suitable control area. Both villages are peri-urban areas located on the outskirts of Yogyakarta City, Indonesia. We divided the sampling area into three categories: large pig farms (a cumulative of three large pig farms), human settlements near pig farms, and human settlements without pig farms. The three sampling areas had almost equal vegetation structure conditions to avoid any bias. We recorded the GPS points of all pig farms and households in the human settlements near pig farms and human settlements without pig farms.

Mosquitoes were collected over a period of 6 weeks (two weeks in each large pig farm and two nights in each household), from the first week of January 2023 to the second week of February 2023, using a total of 50 UV light mosquito traps. Of these, 10 traps were placed at three large pig farms (Farm 1, 2, and 3), which housed 87, 241, and 20 pigs, respectively. The traps in the pig farms were strategically positioned at various locations within the farm, including near the pig pens and feeding areas. Traps were placed at different heights between 0 and 1 m above ground level, with each trap positioned at least 5 m apart to avoid overlap. Traps were placed within 1 m of pig pens, depending on the farm layout.

In addition, 20 traps were placed in human settlements near the large pig farms (120 households in total within Ngestiharjo village), which also contained four small pig farms spread across four households. These traps were set both indoors (e.g. living room) and outdoors (e.g. in front of the house) near the electric socket outlets, and moved between different households every 48 h, covering all 120 households within 6 weeks. The remaining 20 traps were placed in human settlements without any pig farms (120 households in Banguntapan village), with similar placement patterns (both indoor and outdoor) to those in the Ngestiharjo village. The traps in the human settlements were moved every 48 h to new households within the village, ensuring a diverse sample across the study period. The traps in the pig farms were operated continuously for 2 weeks in each pig farm, and mosquitoes were collected every day to avoid drying of the mosquito sample.

We conducted a survey to collect mosquito larvae from the large pig farms. We sought to record factors such as the management of livestock farming, how the farmers manage the cleanliness of the pig farm area (including water presence) to try to elucidate any relationships with mosquito-borne diseases. Mosquito larvae were collected from drinking water drums placed near the animals using plastic pipettes. The larvae were transferred to plastic cups and placed in cages in the Animal Systematic Laboratory, Universitas Gadjah Mada. The larvae were reared to adulthood and identified at the species level using a stereomicroscope and identification guides from the Ministry of Health of the Republic of Indonesia and Rattanarithikul et al. (2010).

The adult mosquitoes were identified by sex and species at the Animal Systematic Laboratory, Faculty of Biology, Universitas Gadjah Mada, Indonesia, based on morphology examined under a stereomicroscope. They were placed in 1.5-ml Eppendorf microtubes and stored at −20 °C.

Damaged and unidentified mosquitoes were identified using a molecular identification method at Ehime University, Japan. Genomic DNA from damaged and unidentified mosquito samples (*n* = 160) was extracted individually from whole mosquitoes using the DNeasy Blood & Tissue Kit (Qiagen, Hilden, Germany). Primer sets for cytochrome *c* oxidase subunit 1 (*cox*1) LCO1490 and HCO2198 (Hebert et al., 2003) were used to identify mosquitoes at the species level. The mastermix was prepared using 0.25 μl of TaKaRa Ex Taq 5 U/μl, 5 μl of 10× Ex Taq Buffer, 4 μl of 25 mM MgCl_2_, 4 μl of 2.5 mM dNTPs, 31.75 μl of molecular grade water, 2 μl of 5 μM forward and reverse primers, and 1 μl of DNA template. The thermal cycling conditions were: initial denaturation at 94 °C for 10 min; 35 cycles of denaturation at 95 °C for 60 s, annealing at 50 °C for 60 s, extension at 72 °C for 60 s; and final extension at 72 °C for 5 min. A total of 160 individual sequences were obtained, and sequence identity searches were performed using BLAST (http://www.ncbi.nlm.nih.gov/BLAST/).

### 2.2 Data analysis

Adult and larval mosquito data were analyzed separately. Mosquito breeding site occurrence in pig farms was assessed using data from larval surveillance. Adult mosquito data were used in community structure, rarefaction, generalized linear model (GLM) and correlation analysis. Community structure across large pig farms, human settlements near pig farms, and human settlements without pig farms was compared and visualized using non-metric multidimensional scaling and permutational multivariate analysis of variance (NMDS) in *vegan* (v.2.6–4) (Oksanen et al., 2020) in R. Mosquito abundance data in the large pig farms, human settlements near pig farms and human settlement without pig farms was used for the NMDS analysis based on the Bray-Curtis dissimilarity. For the community structure analysis using NMDS, we prepared the mosquito data based on the household and pig farm code. The data included the abundance of each mosquito species at the trap site level. To decrease bias regarding the uneven number of traps and nights between the pig farms and human settlements, we computed the mean trap-night abundance and composition between sites. To compare species richness across large pig farms, human settlements near pig farms, and human settlements without pig farms, while adjusting for the influence of different numbers of sampling sites, we used the *iNEXT* package (https://chao.shinyapps.io/iNEXTOnline/) (Chao et al., 2014, 2016) to create rarefaction curves and evaluate whether the samples collected represented true diversity or species richness per sampling location. Rarefaction analysis used the sampling unit (the number of traps multiplied by the site or the household, or the number of large pig farms) to decrease the potential bias of the different number of traps at each sampling site. GLM was carried out in R using the *MASS* package (v.7.3–65) (Venables and Ripley, 2002) to understand the effect of pig farm on mosquito density. Catch per unit effort (CPUE) was calculated by dividing the number of mosquitoes by the total number of traps and the number of days operated at a given location.

The Spearman test was used to examine the correlation between the geographical distance of households to the nearest large pig farm and the total adult female mosquito abundance per household (a significance level of α = 0.05 was used). Maps showing the spatial distribution of mosquitoes in large pig farms and human settlements with pig farms were created using qGIS 3.4 (http://www.qgis.org/) based on GPS coordinates recorded at the human settlements and pig farms.

In addition, we assumed that mosquitoes collected from pig farms are more likely to prefer feeding on pigs or other animals, whereas those collected from human settlements are more likely to prefer feeding on humans. To assess this, we compared the CPUE values of each mosquito species collected from pig farms and human settlements and verified these patterns against their known feeding preferences reported in previous studies.

## 3 Results

### 3.1 Species richness and abundance of mosquitoes

A total of 2253 adult mosquitoes (1663 females and 590 males) were collected from large pig farms, human settlements near pig farms, and human settlements without pig farms. The most abundant genera collected from this study were *Culex* (711 females and 162 males; 38.74%), followed by *Aedes* (391 females and 218 males; 27.03%), and *Anopheles* (306 females and 162 males; 20.77%). Across all three area types, *Cx. tritaeniorhynchus* (*n* = 397) was the most abundant species collected, followed by *Ae. aegypti* (*n* = 333) and *An. vagus* (*n* = 262). These three abundant species are the major vectors for JE, dengue, chikungunya, Zika, and malaria (Table 1). Table 1 indicates the species of mosquito found in the pig farms, human settlements near pig farms and human settlements without pig farms. Large pig farm areas exhibited higher mosquito species diversity and abundance than human settlements. Human settlements located near pig farms showed higher mosquito diversity and abundance than human settlements without pig farms. The average values for mosquito numbers per trap per night were 2.80 for pig farms, 0.82 for human settlements near pig farms, and 0.35 for human settlements without pig farms. Mosquito species diversity was higher in the pig farms with an average of 0.68 species per trap per night, followed by human settlements near pig farms with an average of 0.39 species per trap per night and human settlements without pig farms with an average of 0.22 species per trap per night.

**Table 1:** Abundance of adult mosquito species collected from all sampling areas in Yogyakarta, Indonesia.

Sex	No.	Species	Large pig farms	Human settlement near pig farms	Human settlement without pig farms	Vector for
Female	1	*Aedes aegypti*	29	181	123	Dengue fever, chikungunya, Zika
	2	*Aedes albopictus*	12	15	10	Dengue fever, chikungunya
	3	*Aedes poicilus*	18	2	1	Filariasis
	4	*Anopheles vagus*	241	19	2	Malaria
	5	*Anopheles aconitus*	28	1	0	Malaria
	6	*Anopheles barbirostris*	8	1	0	Malaria, filariasis
	7	*Anopheles kochi*	1	0	0	Malaria
	8	*Anopheles longirostris*	1	0	0	Malaria
	9	*Anopheles maculatus*	2	0	0	Malaria
	10	*Anopheles sundaicus*	1	0	0	Malaria
	11	*Anopheles tesselatus*	1	0	0	Malaria
	12	*Armigeres subalbatus*	22	78	8	Filariasis
	13	*Coquillitidia crassipes*	1	0	0	Rural bancroftian filariasis
	14	*Culex bitaeniorhynchus*	2	0	0	JE
	15	*Culex fuscocephala*	1	3	0	JE
	16	*Culex gelidus*	5	0	0	JE
	17	*Culex hutchinsoni*	127	4	1	No report
	18	*Culex quinquefasciatus*	74	68	13	Filariasis
	19	*Culex tritaeniorhynchus*	381	11	5	JE
	20	*Culex vishnui*	7	2	7	JE
	21	*Ficalbia minima*	65	1	1	No report
	22	*Malaya genurostris*	2	0	0	–
	23	*Mansonia annulata*	1	1	0	Filariasis
	24	*Mansonia uniformis*	29	3	0	Filariasis
	25	*Mimomyia luzonensis*	26	1	0	No report
	26	*Uranotaenia atra*	10	6	0	No report
Male	1	*Aedes* spp.	32	85	101	–
	2	*Anopheles* spp.	116	36	10	–
	3	*Culex* spp.	72	73	17	–
	4	*Armigeres* spp.	1	38	6	–
	5	*Mansonia* spp.	3	0	0	–
Total			1319	629	305	

*Abbreviation*: JE, Japanese encephalitis.

**Fig. 2:**
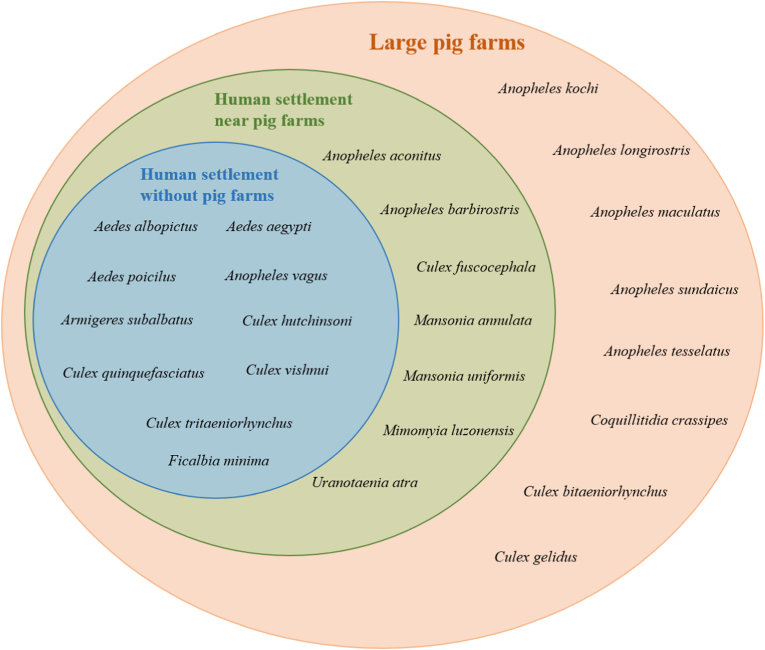
Venn diagram showing species diversity of mosquitoes found in large pig farms (*orange*), human settlements near pig farms (*green*), and human settlements without pig farms (*blue*).

The Venn diagram in Fig. 2 shows the mosquito species collected in the three areas. The habitat distribution of mosquito species in the three areas showed a distinct nested structure. Specifically, large pig farms harbored 26 species, human settlements near pig farms harbored 17 species, and human settlements without pig farms harbored 10 species. Eight species were found only in large pig farms. Human settlements near pig farms exhibited higher mosquito diversity than those without pig farms.

Aggregating by genus, more female mosquitoes from each genus in each sampling area were collected than male mosquitoes (Table 1), excluding *Anopheles* spp. collected from human settlements near pig farms and human settlements without pig farms (Supplementary file 1: Fig. S3). More female mosquitoes were collected from large pig farms than male mosquitoes, especially *Anopheles* and *Culex* spp. In human settlements, we collected nearly equal numbers of female and male *Culex* spp. More female and male *Aedes* spp. mosquitoes were collected from both human settlement settings than from pig farms. Only female *Ficalbia* spp., *Mimomyia* spp., and *Coquilettidia* spp. were collected across all three sampling areas.

From the larval collections, we collected three species of mosquitoes in the same type of container. We found *Cx. quinquefasciatus* (18 females and 15 males), *Ae. albopictus* (13 females and 15 males), and *Ae. aegypti* (5 females and 5 males) in the drum container near the animals in the large pig farms (Supplementary file 1: Fig. S1 and Table S1).

### 3.2 Mosquito community structure

**Fig. 3:**
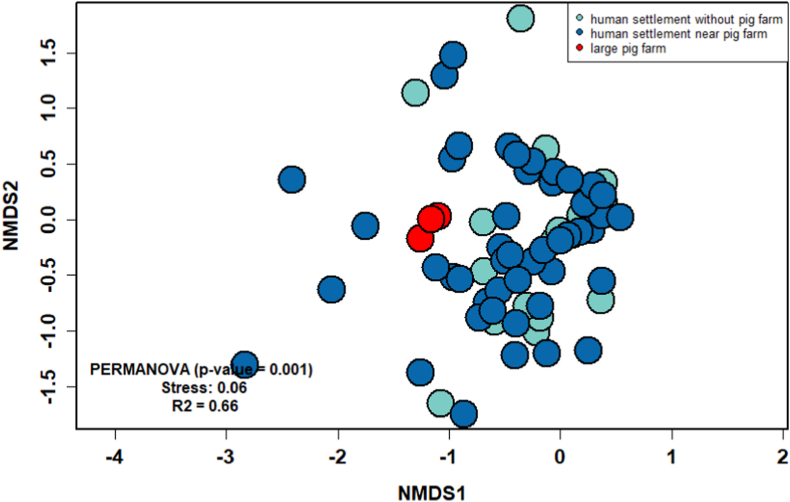
Non-metric multidimensional scaling plot of mosquito community composition and structure showing similarity between pig farms, human settlements near pig farms, and human settlements without pig farms based on the Bray-Curtis dissimilarity matrix. The results of permutational multivariate analysis of variance indicated that mosquito communities differed significantly across the three types of sampling areas (*P* = 0.001, *R*^2^ = 0.66).

Non-metric multidimensional scaling analysis showed that mosquito community structure in human settlements near pig farms and in human settlements without pig farms differed slightly (Fig. 3). The community structure of large pig farms was distinctly different from that of both human settlements near pig farms and human settlements without pig farms. Permutational multivariate analysis of variance revealed significant differences in mosquito community structure across large pig farms, human settlements near pig farms, and human settlements without pig farms (*R*^2^ = 0.66, *P* = 0.001). Pairwise permutational multivariate analysis of variance revealed significant differences for the three mosquito community types (*P* < 0.01). The rarefaction curve showed that species richness was the highest in large pig farm areas, followed by human settlements near pig farms and human settlements without pig farms, after the variation in the number of sampling units was accounted for (Supplementary file 1: Fig. S2).The GLM analysis showed that the presence of pig farms significantly increased mosquito density (*F*_(2, 346)_ = 208.18, *P* < 0.0001).

### 3.3 Effect of geographical distance on mosquito community composition in households near pig farms

**Fig. 4:**
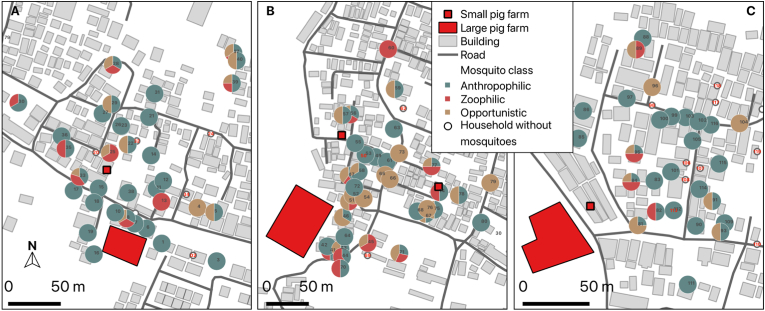
The presence of anthropophilic, zoophilic and opportunistic mosquitoes in the household located near three large pig farms in Ngestiharjo village, Yogyakarta: Farm 1 (**A**), Farm 2 (**B**) and Farm 3 (**C**). Circles represent individual households included in the sampling area, and the numbers inside each circle indicate the household ID used for sampling.

The correlation analysis using pooled data from three large pig farms and human settlement near pig farms revealed no significant correlation between the geographical distance of households to the large pig farms and the diversity or density of female mosquitoes (Spearman’s rho = 0.081, *P =* 0.43; and Spearman’s rho = 0.045, *P =* 0.66, respectively) (Supplementary file 1: Fig. S4). However, community composition analysis showed that zoophilic mosquitoes were found in the human settlement geographically close to the pig farms (Fig. 4).

### 3.4 Host-seeking estimation of mosquitoes through CPUE analysis

Comparison of CPUE values between large pig farms and human settlements (both near pig farms and without pig farms) for each species showed that only *Ae*. *aegypti* had high CPUE values in the human settlements (Table 2). Conversely, 22 mosquito species had higher CPUE values in large pig farms than in human settlements. *Armigeres subalbatus* and *Cx. fuscocephala* had almost equal CPUE values between pig farms and human settlements. The results in Table 2 suggest that *Ae. aegypti* is the only anthropophilic mosquito, while *Ar. subalbatus* and *Cx. fuscocephala* exhibited opportunistic host association behaviour, whilst other species exhibited zoophilic preference.

**Table 2:** CPUE of each mosquito species in the pig farms and the human settlements.

Species	Houses (A)	Pig farms (B)	CPUE human settlements	CPUE pig farms	Tendency	Reference
*Aedes aegypti*	304	29	7.60	2.9	A > B	Anthropophilic (Pruszynski et al., 2020; Sene et al., 2022) and anthropophilic and zoophilic (Khaklang and Kittayapong, 2014)
*Aedes albopictus*	25	12	0.63	1.2	A < B	Opportunistic (Faraji et al., 2014; Khaklang and Kittayapong, 2014; Aure et al., 2016; Pereira-Dos-Santos et al., 2020)
*Aedes poicilus*	3	18	0.08	1.8	A < B	Zoophilic but can feed on humans (Aure et al., 2016)
*Anopheles vagus*	21	241	0.53	24.1	A < B	Zoophilic but can feed on humans (Bashar et al., 2012; Aure et al., 2016; St Laurent et al., 2017; Al-Amin et al., 2023)
*Anopheles aconitus*	1	28	0.03	2.8	A < B	Zoophilic but can feed on humans (Tisgratog et al., 2012; Suwonkerd et al., 2013; Jeyaprakasam et al., 2022)
*Anopheles barbirostris*	1	8	0.03	0.8	A < B	Zoophilic (Bashar et al., 2012; Thankachy et al., 2019; Udom et al., 2021) and zoophilic but can feed on humans (Jeyaprakasam et al., 2022)
*Anopheles kochi*	0	1	0.00	0.1	A < B	Zoophilic (Rattanarithikul et al., 1996) but can feed on humans (Aure et al., 2016; St Laurent et al., 2017)
*Anopheles longirostris*	0	1	0.00	0.1	A < B	Zoophilic and anthropophilic (Keven et al., 2017)
*Anopheles maculatus*	0	2	0.00	0.2	A < B	Zoophilic (Rattanarithikul et al., 1996) but can feed on humans (Aure et al., 2016; Jeyaprakasam et al., 2022; Al-Amin et al., 2023)
*Anopheles sundaicus*	0	1	0.00	0.1	A < B	Zoophilic and anthropophilic (Dusfour et al., 2004; Syafruddin et al., 2020)
*Anopheles tesselatus*	0	1	0.00	0.1	A < B	Zoophilic (Rattanarithikul et al., 1996; St Laurent et al., 2017)
*Armigeres subalbatus*	86	22	2.15	2.2	A = B	Anthrophilic (Intarapuk and Bhumiratana, 2021), anthropophilic but can feed on animals (Pandian and Chandrashekaran, 1980; Khaklang and Kittayapong, 2014; Aure et al., 2016), zoophilic (Tanigawa et al., 2013)
*Coquillettidia crassipes*	0	1	0.00	0.1	A < B	Zoophilic (Aure et al., 2016)
*Culex bitaeniorhynchus*	0	2	0.00	0.2	A < B	Zoophilic but can feed on humans (Thankachy et al., 2019)
*Culex fuscocephala*	3	1	0.08	0.1	A = B	Zoophilic but can feed on humans (Aure et al., 2016; Thankachy et al., 2019)
*Culex gelidus*	0	5	0.00	0.5	A < B	Zoophilic but can feed on humans (Aure et al., 2016) and zoophilic (Thankachy et al., 2019)
*Culex hutchinsoni*	5	127	0.13	12.7	A < B	Zoophilic and anthropophilic (Bashar et al., 2012)
*Culex quinquefasciatus*	81	74	2.03	7.4	A < B	Opportunistic (Garcia-Rejon et al., 2010; Khaklang and Kittayapong, 2014; Aure et al., 2016; McMillan et al., 2019; Thankachy et al., 2019)
*Culex tritaeniorhynchus*	16	381	0.40	38.1	A < B	Zoophilic (Thankachy et al., 2019) but can be anthropophilic (Aure et al., 2016)
*Culex vishnui*	9	7	0.23	0.7	A < B	Zooanthrophilic (Maquart et al., 2022) and zoophilic but can be anthropophilic (Khaklang and Kittayapong, 2014; Aure et al., 2016; Thankachy et al., 2019)
*Ficalbia minima*	2	65	0.05	6.5	A < B	–
*Mansonia annulata*	1	1	0.03	0.1	A < B	–
*Mansonia uniformis*	3	29	0.08	2.9	A < B	Zoophilic (Bashar et al., 2012) and zoophilic (Aure et al., 2016) but can be anthropophilic (Onapa et al., 2007)
*Mimomyia luzonensis*	1	26	0.03	2.6	A < B	Zoophilic (Braima et al., 2017)
*Uranotaenia atra*	6	10	0.15	1.0	A < B	Zoophilic (Toma et al., 2014)

## 4 Discussion

The large pig farms had the highest number of mosquito species, followed by human settlements near pig farms, with human settlements without pig farms showing the lowest number (Table 1). Large pig farms and human settlements near pig farms were hotspots of mosquito species diversity (Fig. 2), potentially increasing the risk of mosquito-borne and zoonotic diseases. In particular, large pig farms showed extremely high species diversity, encompassing all mosquito species found in human settlements near pig farms and those without pig farms. The large pig farms of this study were not located in the countryside but in close proximity to human settlements. As a result, not only zoophilic species but also anthropophilic and opportunistic species inhabited the area (Table 2).

We observed significant differences in mosquito community structure across the three areas (Fig. 3). This result implied that the mosquito community structure between the sampling area in the pig farms, human settlements near pig farms and human settlements without pig farms are significantly different. Rural-adapted mosquitoes, such as *Culex* spp. (e.g. *Cx. tritaeniorhynchus*, *Cx. gelidus)* and *Anopheles* spp., were most abundant on large pig farms, whereas urban-adapted mosquitoes, such as *Ae. aegypti*, were most frequently collected from human settlements (Table 1). These patterns were consistent with other studies. For example, in livestock farms in Sri Lanka, *Culex* spp. were the most common mosquitoes reported (Daluwaththa et al., 2019), with species such as *Cx. fuscocephala*, *Cx. gelidus*, *Cx. tritaeniorhynchus*, and *Cx. vishnui* were showing high abundance in both cattle and pig farms. These species have been implicated in the transmission of various diseases, such as JE, which are prevalent in the region. The higher mosquito populations in these areas may be linked to favorable breeding conditions for mosquitoes near agricultural sites, with a known association between livestock presence and increased vector populations. Additionally, Nguyen et al. (2021b) found that households with pig farms in Vietnam had significantly higher mosquito populations compared to those without, indicating a geographical correlation between pig farming and mosquito abundance. This contrast highlights the importance of understanding local epidemiological conditions, such as livestock presence, to assess the risk of mosquito-borne diseases in different regions.

We collected high numbers of *Cx. tritaeniorhynchus* from large pig farms and some human settlements near pig farms (Table 1). *Culex tritaeniorhynchus* is primary vector of JE (Karthika et al., 2018) and the pigs are considered as amplifying host of JE virus (JEV) (van den Hurk et al., 2009; Impoinvil et al., 2013). Lindahl et al. (2012) found that keeping pigs in urban areas increases the number of JE vectors. The number of pigs was shown to be associated with the increased number of the JE vector *Cx. tritaeniorhynchus* (Lindahl et al., 2012*)*. Based on the Provincial Health Office (2023), the Special Region of Yogyakarta Province had 13 cases of JE in 2014–2021, with 85% of JE cases in Indonesia recorded in the age group of less than 15 years old. Our results suggest that there is potential risk of JE disease transmission in the vicinity of large pig farms and human settlements near pig farms in Yogyakarta due to the high abundance of mosquitoes competent as vectors of JEV (*Cx. tritaeniorhynchus*, *Cx. bitaeniorhynchus*, *Cx. gelidus*, *Cx. fuscocephala*, *Cx. vishnui*). However, in this study, we did not have data on the infection rates of mosquito-borne diseases occurring in the study sites and the vector competence of mosquitoes collected from the sampling sites. We acknowledge this as a limitation to be addressed in future studies by recording pathogen infection rates within the vectors or intermediate hosts. We also had no data on vegetation, human population density and water resources that might affect the mosquito diversity and density found in each sampling site. In this study, we used two different villages for the human settlements near pig farms and human settlements without pig farms. We chose the same land use and topography from both villages by visual inspection to decrease the bias (Supplementary file 1: Fig. S5); however, we could not avoid any additional bias that may be induced due to the unmeasured land use, topography and animal species composition.

Comparison of CPUE values between large pig farms and human settlements (Table 2) showed that among all species, only *Ae. aegypti* had higher CPUE values in human settlements. Based on the CPUE values, our study found that most of the mosquitoes collected exhibited zoophilic preference. More mosquitoes were found in the pig farms than in human settlements, while *Ae. aegypti* was consistently found in high abundance in human settlements, consistent with its well-known anthropophilic behavior (Pruszynski et al., 2020; Wu et al., 2023). In Bangladesh, many species of the genera *Anopheles* and *Culex* were zoophilic (comparable to our data, see Table 2), including *An. vagus*, *An. barbirostris*, and *An. maculatus*, which typically take blood meals more frequently from animals (e.g. cattle, goats, and sheep) than humans (Bashar et al., 2012). In addition, *Cx. gelidus*, *Cx. bitaeniorhynchus*, and *Cx. tritaeniorhynchus* tended to feed on animals (Auerswald et al., 2021). *Culex fuscocephala*, and *Ar. subalbatus* showed no significant difference in CPUEs between pig farms and human settlements. The number of studies on *Cx. fuscocephala* is limited, but according to a study in India, 85 female *Cx. fuscocephala* fed on cattle and only three on humans (Thankachy et al., 2019). Because only four *Cx. fuscocephala* mosquitoes were collected in our study, further studies are needed to confirm their host preferences. *Aedes albopictus* and *Cx. quinquefasciatus* had higher CPUE values in pig farms compared to human settlements. The CPUE value for *Ae. albopictus* in the pig farms *vs* human settlements was different (1.2 > 0.63, respectively). Previous studies reported that *Ae. albopictus* shows opportunistic feeding toward humans and animals (Faraji et al., 2014; Khaklang and Kittayapong, 2014; Aure et al., 2016; Pereira-Dos-Santos et al., 2020). This species was also shown to exhibit opportunistic feeding behavior for a wider range of animals (Delatte et al., 2010) such as humans, reptiles, pigs, dogs, cats, cows and fowls (Ponlawat and Harrington, 2005; Kamgang et al., 2012; Wilson and Sevarkodiyone, 2015; Tsunoda et al., 2022). *Culex quinquefasciatus* has also been reported to have opportunistic preference (Alencar et al., 2012). Our study demonstrated estimating the host-seeking of each mosquito species by comparing the CPUE value from pig farms and the human settlement exhibited parallel findings with the host preference of each mosquito from previous studies (Table 2). The host-seeking estimation using CPUE involves the assumption based on the presence of mosquitoes in human settlement settings (human-preferred) and animal farms (animal-preferred). This assumption may introduce bias, for example, if animals are present in the household. In addition, to accurately estimate the mosquito host seeking using the CPUE value, the human and animal density should be provided. Even though no animal and human host data were used, our results which we used as an inferred proxy for estimating the host-seeking behavior of mosquitoes, were consistent with the previously recorded preferences for each mosquito species (Table 2). Host feeding patterns of mosquitoes can be more accurately estimated by identifying the host species from blood-engorged mosquitoes. However, this requires a large number of mosquitoes to be analyzed to determine the host feeding preference. In addition, blood digestion processes in mosquitoes are quick. Two studies reported that the DNA of the host was no longer detectable after 36–72 h (Ngo and Kramer, 2003; Oshaghi et al., 2006).

Ngestiharjo village, which has large pig farms, had an overall increase in mosquitoes that have a preference for animals compared with Banguntapan village, where no pig farms existed. The zoophilic mosquitoes found in high densities in the pig farms were also found in human settlements near both large and small pig farms (Fig. 4). In addition to large pig farms, small pig farms may have affected the mosquito composition of neighboring human settlements because small pig farms are located behind or next to homes (Fig. 4). In some households we did not collect any mosquitoes. This may be due to the differences in house structure and mosquito bite prevention measures taken in each house.

In large pig farms, female *Anopheles* spp. and *Culex* spp. were more abundant than males. In human settlements, male *Anopheles* spp. were more abundant than female *Anopheles* spp. Some females of *Anopheles* spp. and *Culex* spp. were reported to exhibit animal feeding behavior (Hasegawa et al., 2008; Wilson and Sevarkodiyone, 2015; Maquart et al., 2022; Yeo et al., 2020). Males of *Anopheles* spp. and *Culex* spp. may cluster far from their animal hosts (Paris et al., 2023). Some males of *Anopheles* spp., such as *An. gambiae*, swarmed near larval habitats, female resting places, and human dwellings (Paris et al., 2023). These differences in the habits of males and females of *Anopheles* spp. and *Culex* spp. may have influenced the difference in abundance. However, both males and females of *Aedes* spp. were highly abundant and did not differ in abundance. The habit of male *Aedes* species to congregate on their hosts (humans) (Paris et al., 2023) may have influenced this result (Supplementary file 1: Fig. S3).

Environmental factors can significantly influence mosquito diversity and population dynamics. Humidity, temperature, and precipitation play key roles in shaping mosquito distribution (Brugueras et al., 2020). Pig farm management practices, including sanitation and animal waste handling, and creation of drinking water sites, could provide breeding sites for mosquitoes and thus affect mosquito density (Minakawa et al., 2002; Saul, 2003). Our study supports the above considerations. We found an artificial breeding site in the water container within the pig farm with larvae of three species known to have anthropophilic and opportunistic behavior, *Ae. aegypti*, *Ae. albopictus* and *Cx. quinquefasciatus*. Hoof prints, bathing areas, and watering holes in pig farms may provide additional breeding sites for mosquito larvae (Olson and Meek, 1977; Hansen et al., 1990). The increased number of larval breeding sites within the pig farms could have increased mosquito species diversity and abundance in large pig farms. We collected predominantly zoophilic mosquitoes within the pig farms (Table 2) and *Anopheles* spp. larvae were not found within the large pig farms. This might be due to the typical breeding of *Anopheles* spp. in natural water bodies such as ponds, puddles and rice fields (Munthe et al., 2022). We found only artificial water containers within the large pig farms, which are used by farmers for animal drinking and cleaning. The availability of breeding sites in the landscape influences the flight range of the mosquitoes (Reiter et al., 1995). *Anopheles* spp. could reach the maximum flight distance of 4.5 km when blood-fed (Kaufmann and Briegel, 2004) to search for potential breeding sites. Our result may indicate that *Anopheles* spp. mosquitoes come to the pig farm to feed on the animal due to their zoophilic preference and then fly away in order to find suitable breeding sites outside the pig farm. Future studies need to be conducted to understand the flight distance of *Anopheles* spp. from the breeding site to the farm where the adult mosquitoes feed on animal blood.

## 5 Conclusions

Mosquito community structure of the three sampling areas was significantly different. Species diversity and abundance were higher in large pig farms than in human settlements near pig farms and human settlements without pig farms in the peri-urban area of Yogyakarta, Indonesia. Zoophilic mosquitoes (e.g. *Anopheles* spp. and *Culex* spp.) were most abundant in pig farms, whereas anthropophilic mosquitoes (e.g. *Ae. aegypti*) were abundant in human settlements. Some zoophilic mosquitoes were found in human settlements geographically close to pig farms. The presence of pig farms near human settlements increased the diversity and density of mosquitoes in those settlements, including high densities of JE vectors such as *Cx. tritaeniorhynchus*. This highlights a potential risk of mosquito-borne diseases in these areas. To mitigate this risk, local governments should consider relocating pig farms away from human settlements or, if relocation is not feasible due to logistical, regulatory, financial, or socio-economic constraints, implement improved farm management practices. Such measures include covering stagnant water sources, regularly eliminating potential breeding sites, applying insecticides to livestock, and cleaning animal enclosures. New alternative jobs or investment in community development should be provided by the government if the relocation leads to job losses to decrease the potential negative socio-economic consequences of relocation. Preventive actions, such as routinely draining standing water and covering water storage containers, are also advised to help reduce vector populations and disease transmission risks.

## Ethical approval

This study was conducted according to the Ethical Principle approved by Medical and Health Research Ethics Committee Faculty of Medicine, Public Health and Nursing Universitas Gadjah Mada, Indonesia (KE/FK/0002/EC/2023).

## CRediT authorship contribution statement

**Atikah Fitria Muharromah:** Conceptualization, Investigation, Visualization, Writing - original draft. **Raden Roro Upiek Ngesti Wibawaning Astuti:** Supervision, Writing - review & editing. **Kozo Watanabe:** Conceptualization, Supervision, Writing - review & editing.

## Funding

This study was financially supported by the Japan Society for the Promotion of Science (JSPS) Core-to-Core Program B. Asia-Africa Science Platforms (JPJSCCB20240008) and the JSPS Bilateral Joint Research Projects (120249930). It was also supported by the Ministry of Education, Culture, Sports, Science and Technology, Japan (MEXT) to a project on Joint Usage/Research Center – Leading Academia in Marine and Environment Pollution Research (LaMer).

## Declaration of competing interests

The authors declare that they have no known competing financial interests or personal relationships that could have appeared to influence the work reported in this paper.

## Data Availability

All data generated or analyzed during this study are included in this published article and its supplementary files. The newly generated mosquito sequences are available in the GenBank database under the accession numbers PQ219079-PQ219238.
