# Supplementary material for: Impact of peri-urban pig farms on mosquito community structure in Yogyakarta, Indonesia
Source: Curr Res Parasitol Vector Borne Dis. 2025 Aug 20;8:100310. doi: 10.1016/j.crpvbd.2025.100310 (PMC12423713; doi:10.1016/j.crpvbd.2025.100310)
Supplement: Multimedia component 1 [file mmc1.pdf]

Supplementary file 1

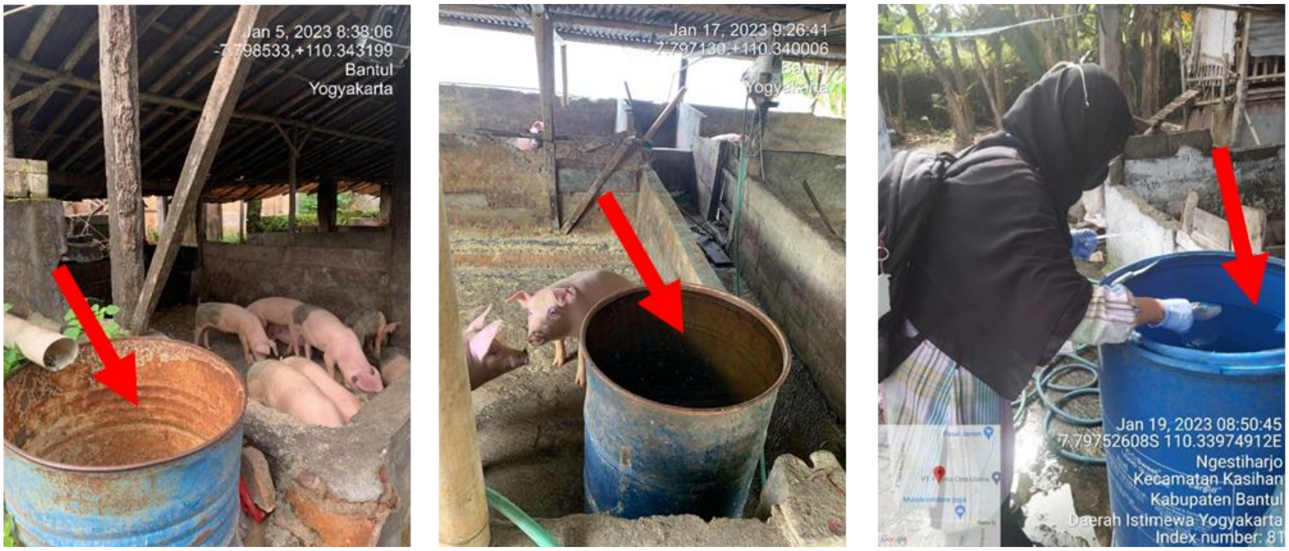

Supplementary Figure S1. Mosquito larvae found in the drinking water of the swine inside the livestock area.

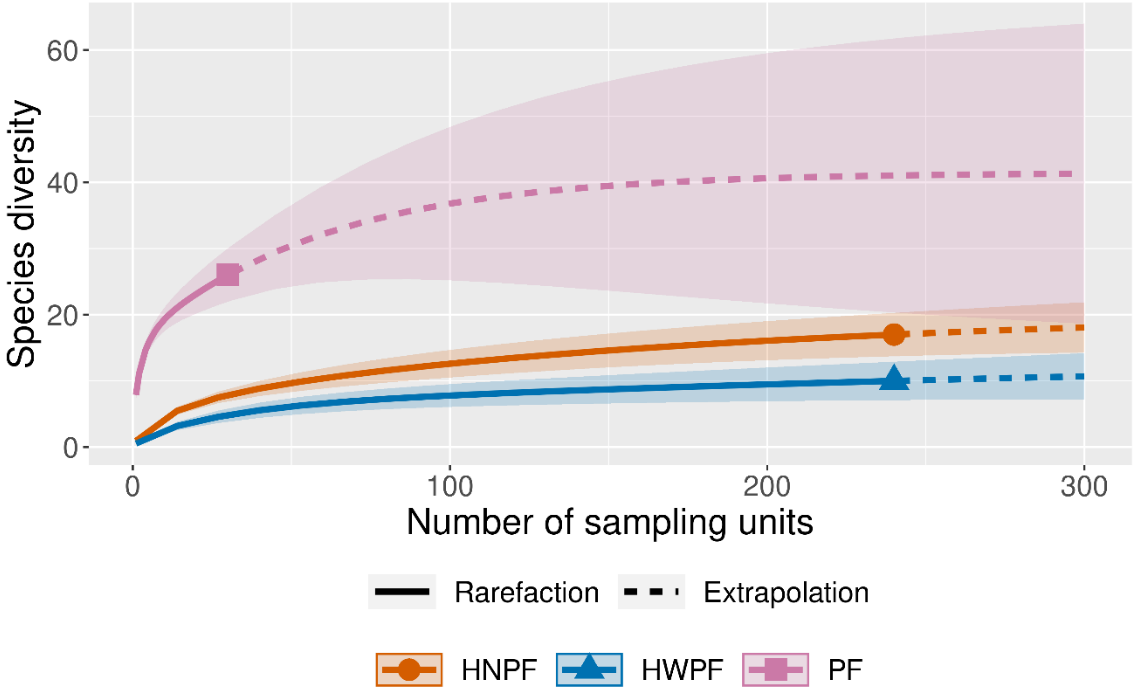

Supplementary Figure S2. Rarefaction curves based on the mosquito diversity and the total number of individuals in each sampling site. *Abbreviations*: PF, pig farm; HNPF, human settlement near pig farm; HWPF, human settlement without pig farm. Solid line represents rarefaction/interpolated data, the dashed line represented extrapolated data. The shaded area for each line indicates 95% confidence intervals.

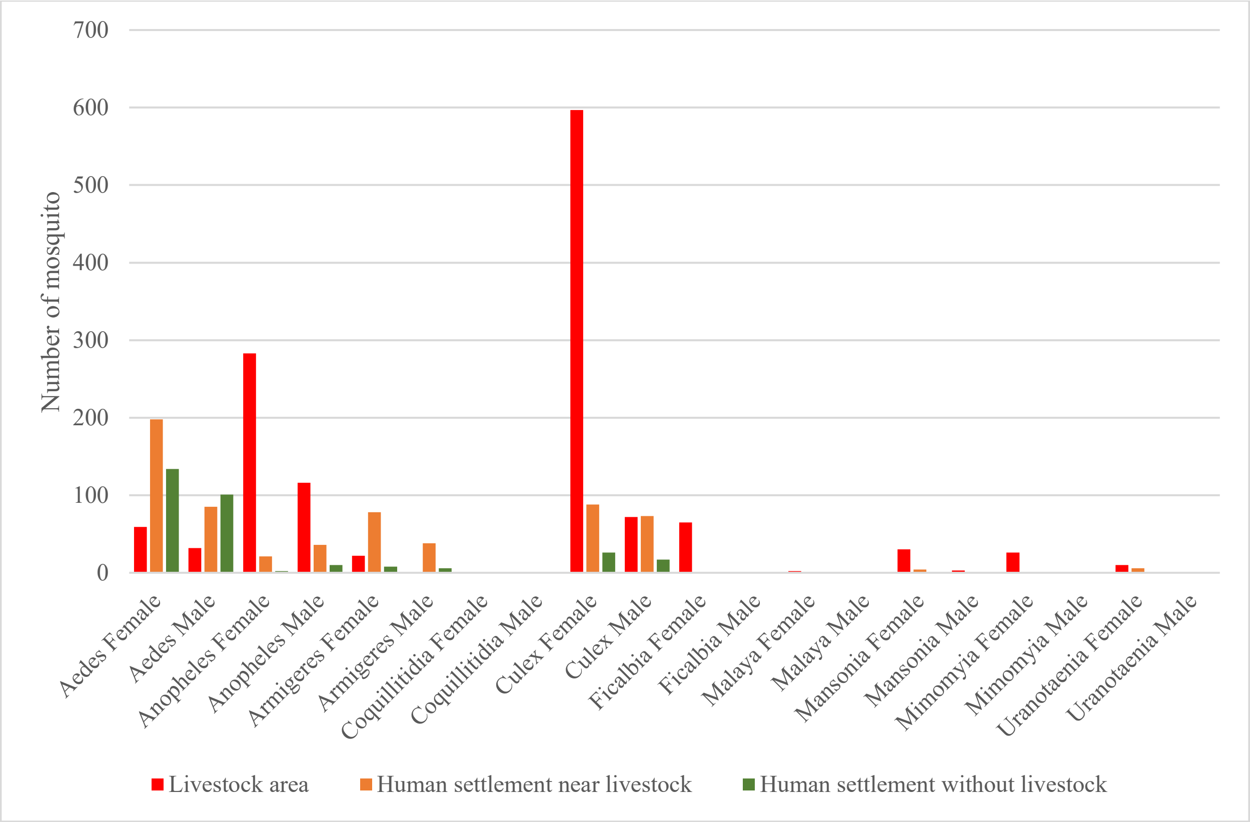

Supplementary Figure S3. Female and male mosquitoes collected from livestock areas, human settlements near livestock, and human settlements without livestock.

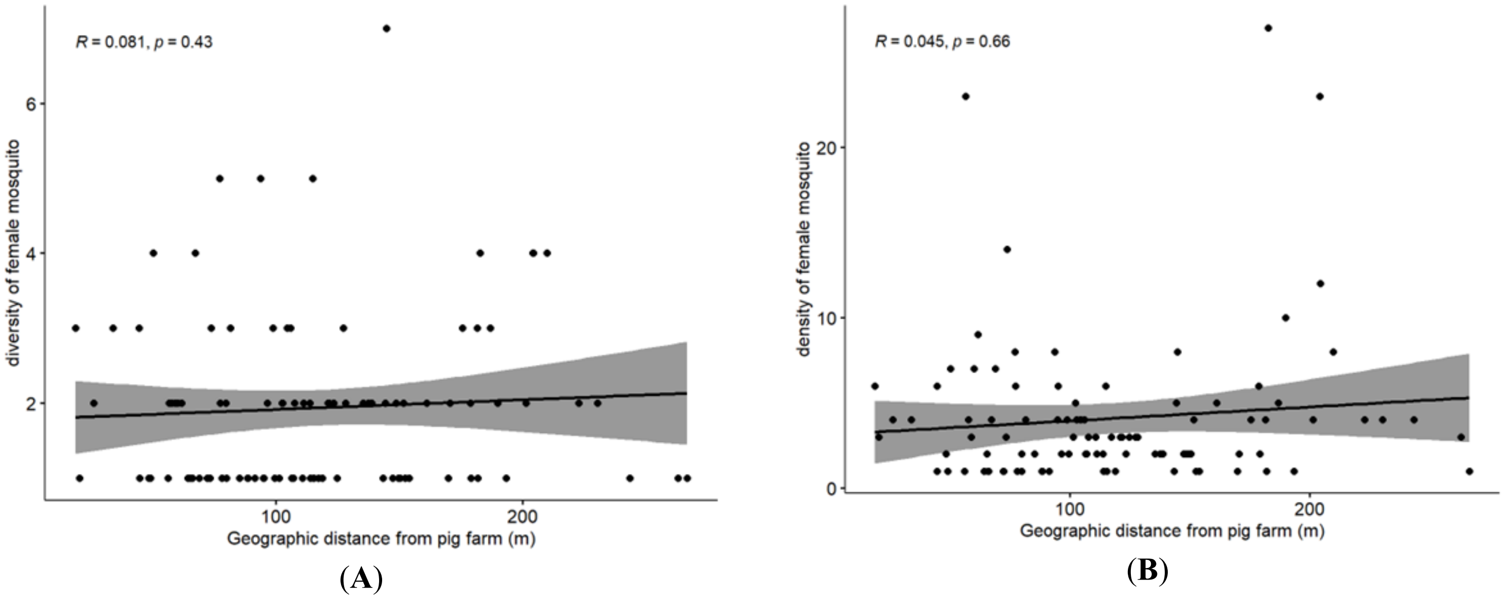

Supplementary Figure S4. Correlation graph between the geographical distance of each household with large pig farms and the diversity (**A**) and density (**B**) of mosquitoes in the household.

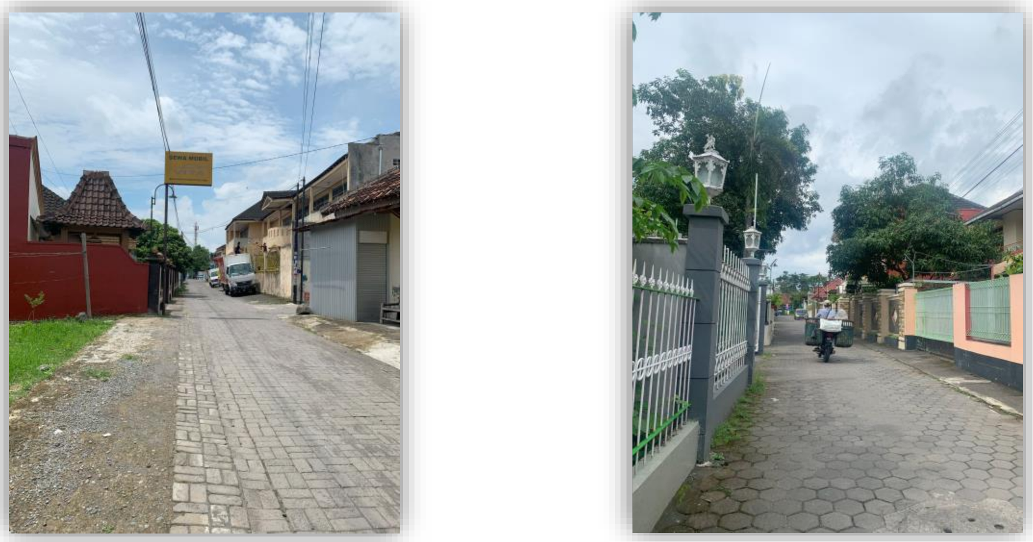

(A) **(B)**

Supplementary Figure S5. The sampling site in Ngestiharjo Village (**A**) and Banguntapan Village (**B**).

Supplementary Table S1. Mosquito diversity and abundance for the larvae collected in a water container within a pig farm

Sex	Species	Total no. of individuals
Female	*Aedes aegypti*	5
	Aedes albopictus	13
	Culex quinquefasciatus	18
Male	*Aedes aegypti*	5
	Aedes albopictus	15
	Culex quinquefasciatus	15
